# Open Partial Nephrectomy for Pediatric Renovascular Hypertension

**DOI:** 10.1100/tsw.2009.40

**Published:** 2009-04-28

**Authors:** Matt S. Ashley, Gregory Moneta, Siamak Daneshmand

**Affiliations:** Section of Urologic Oncology, Division of Urology and Renal Transplantation, Division of Vascular Surgery, Oregon Health and Science University, Center for Health and Healing, Portland, OR 97239, USA

**Keywords:** renovascular hypertension, nephrectomy, pediatrics, renin, urologic surgical procedures

## Abstract

Pediatric renovascular hypertension is typically managed with revascularization, angioplasty, or radical nephrectomy. We describe the case of a 13-year-old boy with medically refractory renovascular hypertension who presented to our institution after a failed arterial bypass. Subsequent angiography and renin sampling of the segmental renal veins suggested that the lower pole of the kidney was affected exclusively by the relative hypoperfusion. We proceeded with an open partial nephrectomy in order to excise the affected region of the kidney, while preserving maximum renal function. The patient was normotensive off all antihypertensive medication and without complications 8 months postoperatively. We believe that partial nephrectomy is a reasonable treatment for children with renovascular hypertension secondary to segmental hypoperfusion, and it should be considered as alternative therapy.

## INTRODUCTION

Between 5 and 25% of all pediatric hypertension is renovascular in etiology. This is in contrast to the adult population, in which only 1% of hypertensive patients have an identifiable renovascular anomaly[1]. Renovascular hypertension in children is most commonly the result of fibrodysplastic disease and can affect any level of the renal arterial circulation.

Historically, pediatric renovascular disease was successfully managed with radical nephrectomy or arterial bypass. However, there are now a number of treatment options available depending on the location of the lesion. Involvement of the main renal artery indicates revascularization by balloon angioplasty or arterial graft. If the disruption affects the segmental circulation exclusively, partial nephrectomy can also yield excellent results[2]. This procedure usually requires localization of the lesion through segmental vein renin sampling. Radical nephrectomy is still pursued when the patient's anatomy is not amenable to a nephron-sparing approach.

## CASE REPORT

A 13-year-old boy presented to a community hospital with a 3- to 4-week history of severe headache and vomiting. He was found to have uncontrolled hypertension of unknown duration with systolic pressures averaging 220 mmHg. A daily regimen of Lisinopril and Amlodipine decreased systolic and diastolic pressures to 140 and 100 mmHg, respectively. The patient also took Clonidine as needed for breakthrough symptoms. A spiral CT scan showed 99% stenosis of the left main renal artery involving a 15-mm segment just distal to the osteum. A left accessory artery to the lower pole of the kidney was also reported as 99% stenosed with involvement of the osteum.

The options for treatment were balloon angioplasty and complex arterial bypass. Due to the unfavorable morphology of the lesion, the patient underwent bypass of the left main renal and lower accessory arteries using a right hypogastric artery graft.

Antihypertensive medications were not resumed after the procedure, and the patient's systolic pressure was controlled between 120 and 140 mmHg for a period of approximately 2 weeks. At this time, the patient presented again with headache and elevated blood pressure. A CT scan demonstrated a fully patent graft to the middle renal artery, but no flow to the inferior renal artery and likely thrombosis. Decreased enhancement was also noted in the lower pole of the kidney (Fig. 1).

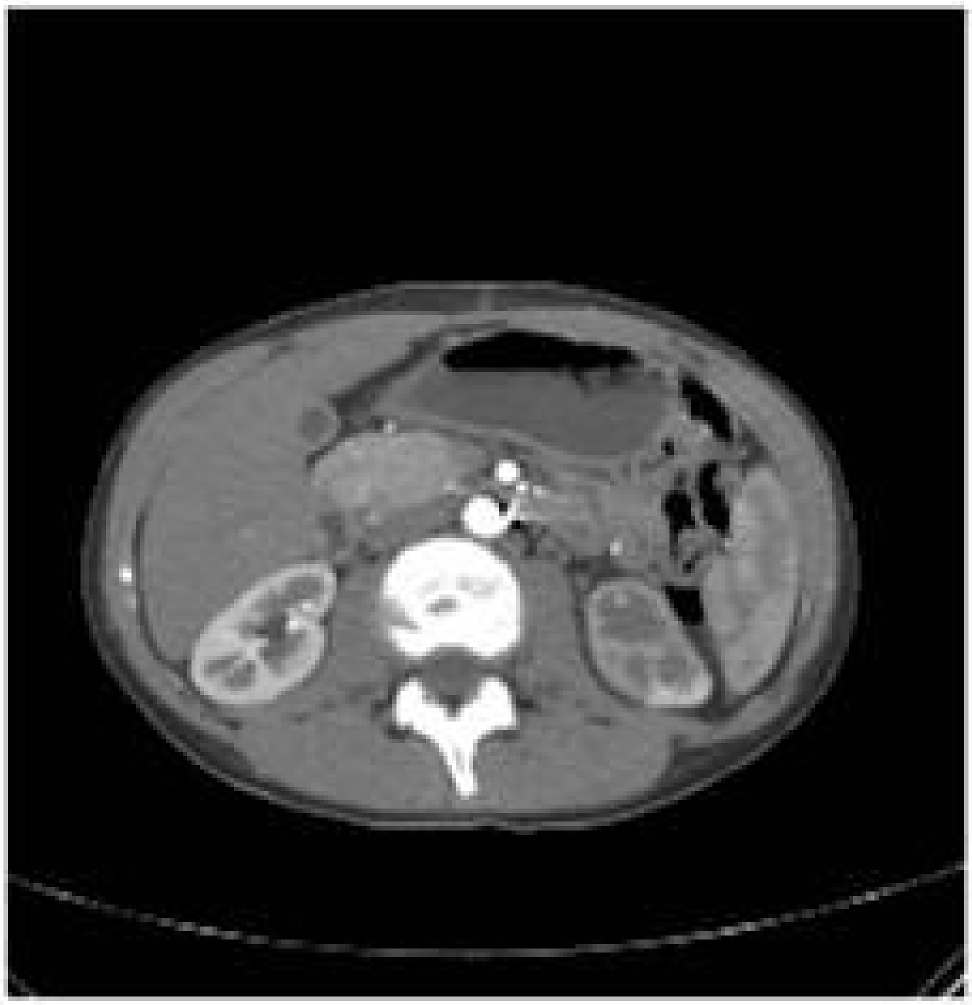

**FIGURE 1**. Enhanced arterial phase CT scan demonstrating left lower pole hypoperfusion.

The patient was on a daily antihypertensive regimen of Amlodipine, Lisinopril, and Metroprolol, with Clonidine as needed. A renal angiogram was performed that included renin sampling from the vena cava, main renal vein, and segmental renal veins (Fig. 2). Imaging showed an occluded bypass graft of the inferior accessory artery and resultant hypoperfusion of the lower pole of the kidney. Renin levels were 9–10 ng/ml/h in the main and lower branches of the renal vein, and 6 ng/ml/h in the upper pole segment, indicating segmental hypoperfusion. The lower pole amputation was performed under warm ischemia using the angiogram as a guide.

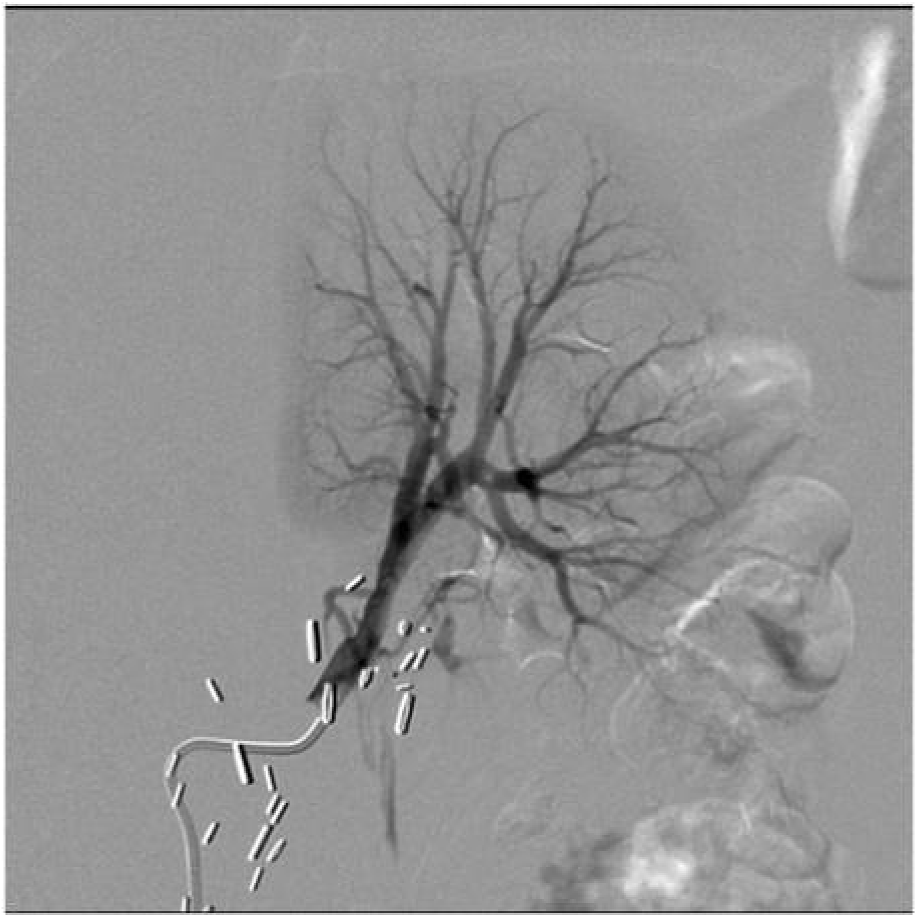

**FIGURE 2**. Renal arteriogram showing delayed filling in the lower pole of the kidney.

During the patient's postoperative hospital stay, blood pressures averaged 110 mmHg systolic and 65 mmHg diastolic. He remains completely normotensive with normal renal function 8 months postoperatively without need for any antihypertensive medications.

## DISCUSSION

Standard treatment for renovascular hypertension includes balloon angioplasty or renal revascularization. Occasionally, total nephrectomy is required in complex cases. Partial nephrectomy is an option if the hypoperfusion can be localized to a resectable segment of the kidney[3]. In this case, given the patient's age and our ability to predict the region of compromised blood flow, we opted to preserve as much kidney parenchyma as possible in order to minimize the impact on renal function.

Due to the limited follow-up, we are unable to assess the patient's long-term blood pressure control. Moreover, international experience with partial nephrectomy for pediatric renovascular hypertension is extremely limited. However, Tash and colleagues report a series of four patients who underwent segmental renal vein sampling and partial nephrectomy, all of whom remain normotensive at a mean follow-up of 10 years[2]. Additionally, Candel-Pau et al. published the case of a child with renovascular hypertension secondary to segmental renal infarction treated with partial nephrectomy who remains normotensive 6 years postoperatively[4].

Partial nephrectomy is a reasonable treatment for children with renovascular hypertension secondary to segmental hypoperfusion, and should be considered as alternative therapy.
